# Clinical Nomograms for Predicting the Overall Survival and Cancer-specific Survival of patients with Ovarian Carcinosarcoma patients after Primary Surgery

**DOI:** 10.7150/jca.63224

**Published:** 2021-10-25

**Authors:** Fang Ren, Shengtan Wang, Feifei Li, Jian Gao, Haoya Xu, Xianli Li, Liancheng Zhu

**Affiliations:** 1 Department of Obstetrics and Gynecology, Shengjing Hospital of China Medical University, Shenyang 110004, Liaoning, China.; 2 Department of Gynecology, Hainan General Hospital, Hainan Affiliated Hospital of Hainan Medical University, Haikou 570011, Hainan, China.; 3 Department of Gynecology, Shandong Provincial Hospital Affiliated to Shandong First Medical University, Jinan 250021, Shandong, China.

**Keywords:** Ovarian carcinosarcoma, Nomogram, Overall survival, Cancer-specific survival, Validation

## Abstract

**Background:** At present, there is no clinical prediction model for ovarian carcinosarcoma (OCS) that is based on a large sample of real data. This study aimed to construct nomograms using data extracted from the Surveillance, Epidemiology, and End Results (SEER) database that can be used to predict the overall survival (OS) and cancer-specific survival (CSS) of patients with OCS and further guide the choice of clinical treatment.

**Methods:** We selected 2753 cases of OCS from the SEER database from 1998 to 2016. Patients were randomly divided in a 7:3 ratio into a training cohort (n = 1929) and a validation cohort (n = 824). Cox analysis was used to select prognostic factors for OS and CSS, and nomograms were then established. The performance of nomogram models was assessed using the concordance index, the area under the receiver operating characteristic curve, calibration curves, and by decision curve analysis. Data from 21 OCS patients at Shengjing Hospital from 2001 to 2021 were collected for external verification. Kaplan-Meier curves were plotted to compare survival outcomes between subgroups.

**Results:** Nomograms based on independent prognostic factors showed good predictive power and clinical practicality. Internal and external validation indicated that the nomograms performed better than staging and grading systems. Significant differences were observed in the survival curves of different risk subgroups.

**Conclusions:** The developed nomograms will enable individualized evaluation of the OS and CSS, thus guiding the treatment of patients with OCS.

## Introduction

Ovarian cancer is a common malignant tumor of the female reproductive system. The morbidity and mortality of ovarian cancer are the third-highest and highest, respectively, among tumors of the female reproductive system 1. More than 75% of ovarian cancer patients are diagnosed at the advanced stage, and the 5-year survival rate is less than 45%. The most common pathological type of ovarian cancer is epithelial ovarian cancer 2,3, which is classified according to serous and mucinous histologies and which accounts for 90% of ovarian cancers^4^. Ovarian carcinosarcoma (OCS) is a rare gynecological malignancy accounting for 1-4% of ovarian malignancies 5.

OCS is a malignant mixed Müllerian tumor, which consists of both carcinomatous and sarcomatous components. Compared with other types of ovarian malignancies, OCS tends to occur in older women and is often detected at an advanced stage, when the prognosis is poor. At present, the staging systems of the American Joint Committee on Cancer and the International Federation of Gynecology and Obstetrics are most frequently used to evaluate the prognosis of patients with ovarian malignancy. However, a study reported that the prognosis of patients with OCS was worse than that of patients with high-grade serous ovarian cancer at the same stage 6. This result suggests that the staging system is not sufficiently accurate to reflect the prognostic differences between OCS and other pathological types of ovarian cancer. Therefore, a comprehensive model is needed to predict the prognosis of patients with OCS and to guide the choice of clinical treatment. However, due to the rarity of OCS, few related studies have been reported, and an effective and convenient tool, based on a large sample of real data, to evaluate the prognosis of patients with OCS is lacking.

A nomogram is a statistical prediction tool with the ability to integrate key predictors. It is widely used to quantify risk and evaluate prognosis in multiple cancer types 7. However, to our knowledge, no clinical prediction model based on a large sample of real data exists for patients with OCS. Therefore, this study aimed to use data extracted from the large Surveillance, Epidemiology, and End Results (SEER) database to build nomograms to predict the prognosis of OCS and to guide the selection of clinical treatment options.

## Materials and methods

### Ethics Approval and Informed Consent

Ethical approval for the use of patient data for external validation in this study was obtained from the Clinical Research Ethics Committee of Shengjing Hospital of China Medical University (Ethical code: 2021PS530K), and all patients provided signed informed consent in accordance with the Declaration of Helsinki.

### Data source

Medical records of patients with OCS were obtained from the SEER database, which contains data of cancer patients from 18 regional registries, covering approximately 34.6% of the total population in the United States. Relevant information was retrieved applying SEER*Stat software version8.3.6 (https://seer.cancer.gov/seerstat/) (account ID: 19731-Nov2019).

### Data extraction

From 1988 to 2016, patients diagnosed as primary OCS and had clear staging, pathological differentiation and surgical conditions were included in the study. The inclusion site code was C56.9-Ovary, and the histological code was 8800, 8801, 8802, 8804, 8805, 8810, 8814, 8840, 8850, 8851, 8854, 8890, 8891, 8896, 8900, 8901, 8902, 8920, 8921, 8930, 8931, 8933, 8935, 8936, 8950, 8951, 8980, 8981, 9044, 9120, 9180, 9220, 9260, 8575, according to the International Classification of Tumor Diseases, Third Edition (ICD-O-3).

Inclusion and exclusion criteria: (1) Patients with pathologically confirmed primary OCS were included, and patients with multiple tumors or secondary tumors were excluded; (2) OCS patients with unknown histological type (NOS patients) were excluded; (3) Year of diagnosis, age, race, marriage and insurance were included; (4) Since grade and stage were known to be prognostic factors, patients with complete information on the stage and histological grade were included; and the patients with incomplete above information were excluded; (5) The patients who had undergone surgery on primary lesion with clear and complete records on surgical method, surgical of lymph nodes, tumor size and residual lesions were included, and the patients who had not undergone surgery were excluded; (6) Patients with incomplete follow-up time or unknown death status or less than 1 day survival were excluded. Variables for this study included: year of diagnosis, age at diagnosis, race, insurance, marriage, laterality, tumor size, preoperative serum CA125 level, surgery for primary lesions, regional lymph nodes (LNs) dissected, number of examined LNs, number of positive LNs, grade, stage, residual lesion size, radiotherapy, chemotherapy, organ metastasis which referred to liver, lung, bone, and brain. After the screening, a total of 2753 patients were included in this study. The initial operation of OCS was taken as the starting point of follow-up, and the end point was death or follow-up until December 31, 2015.

The clinical records of 21 patients who underwent surgery in the Department of Gynecology, Shengjing Hospital of China Medical University from 2001 to 2021 and were pathologically diagnosed as having OCS were retrospectively analyzed. Inclusion criteria: 1. Surgery was primary surgery; 2. The tumor was primary, and the postoperative pathological diagnosis was confirmed as OCS. The clinical data and postoperative follow-up data were complete. The cause of death was cancer-specific death. The end of follow-up was May 25, 2021.

### Statistical methods

X-tile software v3.6.1 (Yale University, New Haven, Connecticut, USA) 8 was used to ascertain the optimal cut-off points for age, tumor size, the number of lymph node examined and number of positive lymph nodes. Patients enrolled in our study were randomly (7:3) divided into a training cohort (n=1929) and a validation cohort (n=824). The primary end points were overall survival (OS) and cancer-specific survival (CSS). Categorical variables were shown as frequencies and proportions. The comparison of clinicopathological characteristics between the training and validation cohorts was performed using a chi-squared test.

Significant prognostic factors were further identified in multivariate analysis from the Cox proportional hazards model. Then, the nomograms associated with OS and CSS were constructed incorporating the final risk factors. The performance of the nomogram was validated internally in the training cohort and externally in the validation cohort. Harrell's concordance index (C-index) ranging from 0.5 to 1.0 was used to evaluate the discriminative abilities of the nomograms. Calibration curves (1000 bootstrap resamples) were generated to test the consistency between the predicted and actual 3- 5- 10-and 13-year OS and CSS.

**Figure 1:**
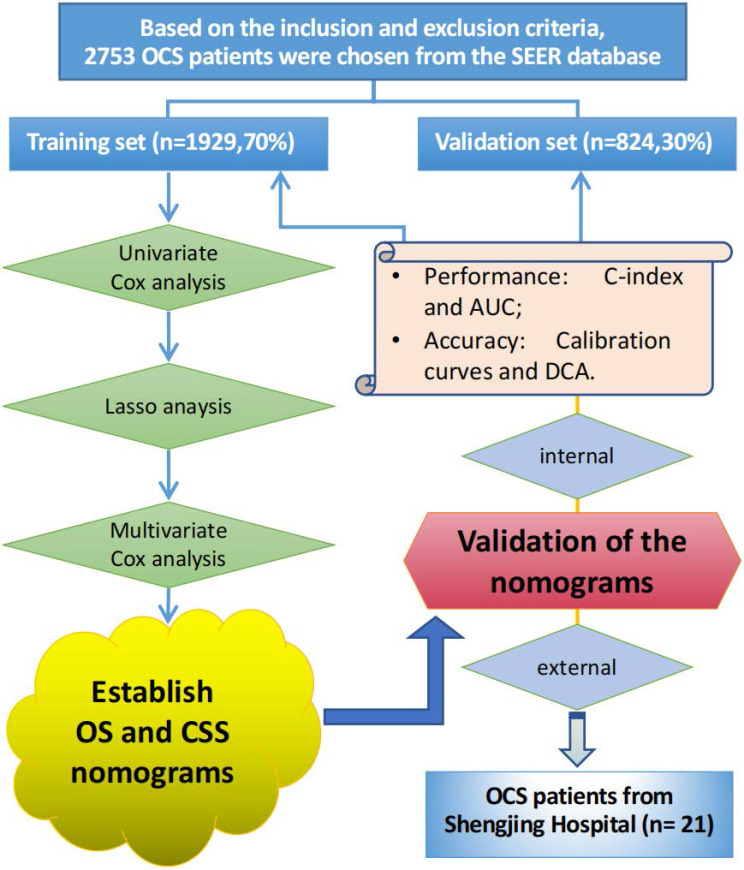
The flowchart of the study.

The traditional ROC curve analysis is a statistical abstraction, which can't directly provide clinical value information 9. In contrast, decision curve analysis (DCA) is more effective in evaluating the clinical utility of the model. Clinical practicability is an important indicator to determine whether the prediction model can be used in clinical work and benefit patients. However, few studies have used this new method to evaluate the net income of the forecasting model. In this study, in addition to observing the c-index, calibration curves and AUC of training cohort and validation cohort to prove that our nomograms have excellent performance and better clinical prediction ability, we also use DCA curve to evaluate the clinical practicability of our model. Emerging as a new method, DCA was applied to evaluate the latent value of the nomograms 10. The clinical records of 21 patients with pathologically diagnosed OCS who underwent surgery in the Department of Gynecology at Shengjing Hospital of China Medical University from 2001 to 2021 were collected for external validation of the model. The flow chart of the study is shown in **Figure 1.**

All statistical analyses were performed with R software (Version 1.2.1335; http://www.r-project.org/) and SPSS (version 25.0, SPSS, Chicago, IL, USA). A p value of < 0.05 was considered statistically significant.

## Results

### Patient characteristics

According to the inclusion and exclusion criteria, a total of 2753 patients diagnosed with OCS were selected from the SEER database. These patients were randomly (7:3) divided into a training cohort (n=1929) and a validation cohort (n=824). There was no significant difference in the included variables between the two groups (all P > 0.05), details in **Table 1.**

### Construction of the prognostic nomograms for OS and CSS

#### Univariate and least absolute shrinkage and selection operator (LASSO) analysis

**Figure 2:**
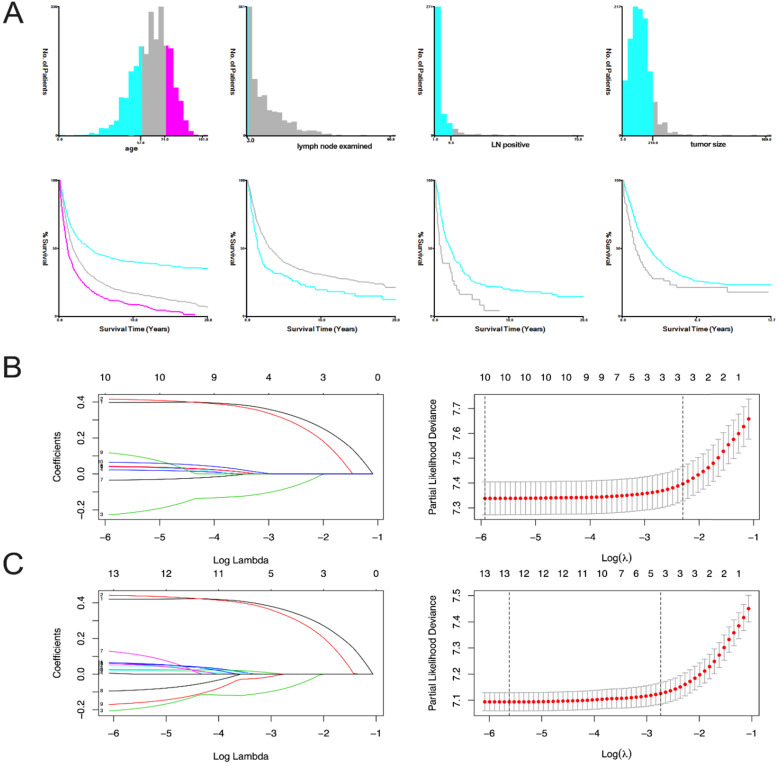
** X-tile stratification and LASSO analysis. (A)** The optimal cut-off points for age were 57 and 74, the p-value of corresponding Kaplan-Meier curve was <0.01; The optimal cut-off points of examined lymph nodes were 3, the p-value of corresponding Kaplan-Meier curve was 0.00014; The optimal cut-off points of positive lymph nodes were 9, the p-value of corresponding Kaplan-Meier curve was 0.00063; The optimal cutoff points of tumor size were 210mm, the p-value of corresponding Kaplan-Meier curve was 0.0002. The LASSO regression used to select prognostic factors for OS and CSS: **(B)** LASSO coefficient profiles of variables for OS; LASSO analysis identified 10 variables for OS. **(C)** LASSO coefficient profiles of variables for CSS; LASSO analysis identified 12 variables for CSS.

The variables are stratified according to the cut-off points which were ascertained by X-tile software v3.6.1: age: ≤56 years, 57-74 years and 75-101 years; tumor size: ≤21 mm and ≥22 mm; number of lymph nodes examined: ≤3 and ≥4; and number of positive lymph nodes: ≤9 and ≥10 **(Figure 2A)**.

In OS analysis, all 21 variables were analyzed by univariate and the least absolute reduction and selection operator (LASSO) method, 11 variates (race, tumor size, CA125, liver metastasis, bone metastasis, insurance, year of diagnosis, chemotherapy, radiation, brain metastasis, lung metastasis) with *P*>0.05 were excluded **(Table 2).** The remaining 10 variables were included in the multivariate analysis **(Figure 2B).** We did CSS analysis in the same way, 9 variables (laterality, race, insurance, liver metastasis, lung metastasis, bone metastasis, radiation, brain metastasis and chemotherapy) with *P*>0.05 were excluded **(Table 2).** The remaining 12 variables were included in the multivariate analysis** (Figure 2C).**

#### Multivariate analysis

All the 10 variables conforming to the OS analysis were included in the multivariate Cox analysis **(Table 2).** Variables with *P*<0.05 was regarded as the independent risk factor of OS, which include stage, grade, age at diagnosis, LN status and residual lesion size.

In the same way, we did multivariate Cox analysis for CSS **(Table 2).** Variables with *P* < 0.05 was regarded as the independent risk factor of CSS, which include stage, grade, age at diagnosis, regional LNs dissected, tumor size, residual lesion size and marital status.

#### Nomogram Construction

**Figure 3:**
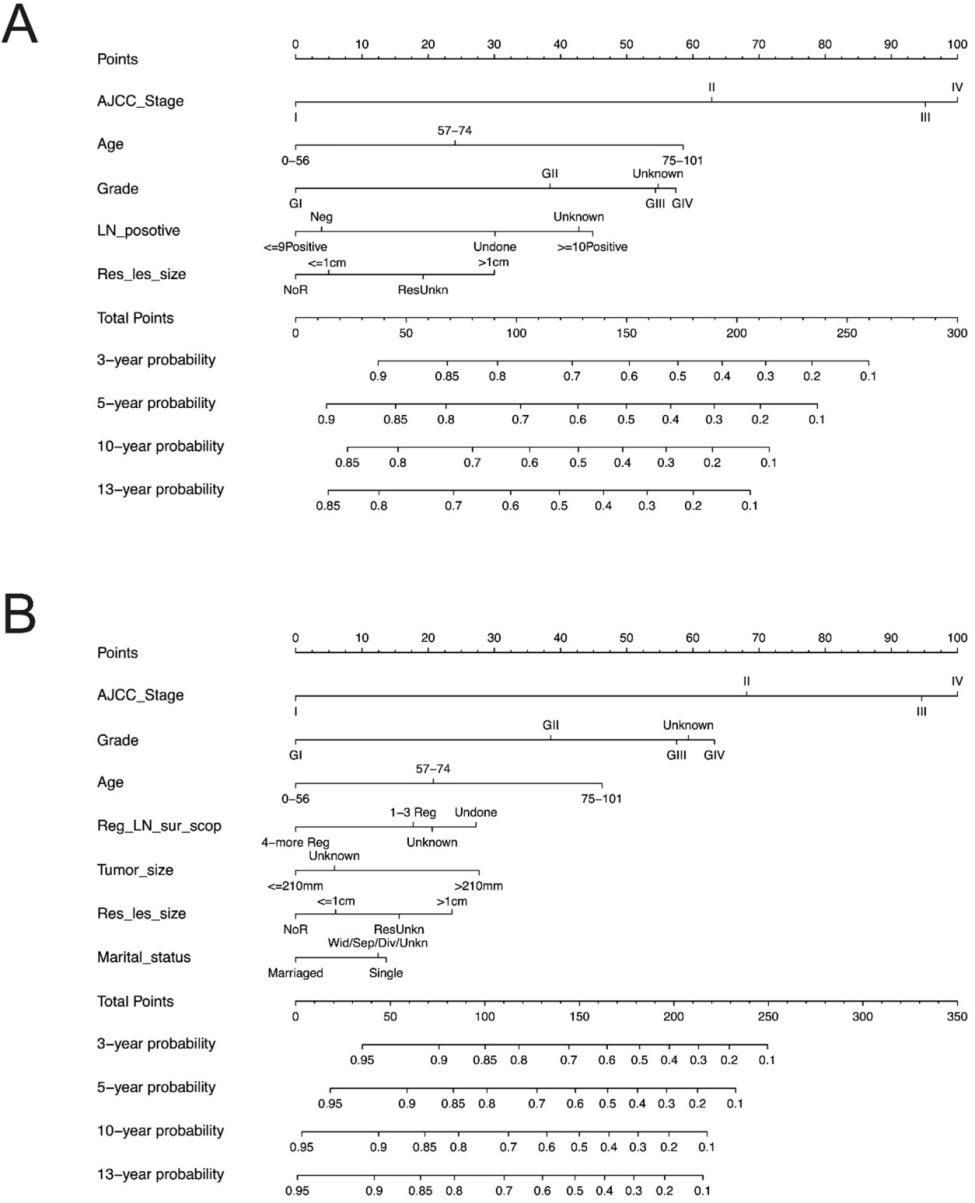
** Predictive nomograms. (A)** Nomogram for predicting 3, 5, 10 and 13-year OS. **(B)** Nomogram for predicting 3, 5, 10 and 13-year CSS. OS: overall survival; CSS: cancer-specific survival; AJCC: American Joint Commission on Cancer. LN: lymph node; Res_les_size: residual lesion size; Reg_LN_sur_scop: regional lymph node surgery scope.

We constructed the nomogram based on above independent prognostic factors for OS and CSS, respectively. The nomogram was displayed for predicting the 3- 5-10- and13-year OS and CSS **(Figure 3).** The different subtypes of each independent prognostic factor were projected onto the score scale to obtain the score for each item. The scores corresponding to independent prognostic factors were added to obtain the total score. A vertical line was drawn down on the total score scale to obtain the 3- 5-10- and 13-year OS and CSS. The higher the total score, the worse the prognosis. According to the patient information, this nomogram could obtain the individualized prediction of OS and CSS, which improves the accuracy and efficiency of the prediction.

Each subgroup variable was assigned a corresponding score for the construction of this nomogram. A score system was used to assign a score of 0 to 100 for each subgroup variable according to its contribution. These scores were added across enrolled variables to generate total scores on the bottom scales, which were then transformed to predict the corresponding OS and CSS.

The nomograms demonstrated that the stage was the largest contributor to prognosis, followed by grade, age at diagnosis, number of positive LN and residual lesion size which also showed a moderate effect on OS **(Figure 3A).** Nomogram of CSS demonstrated that grade, age at diagnosis, regional LNs dissected, tumor size, residual lesion size and marriage had a moderate effect on CSS **(Figure 3B).**

### Nomogram validation/Performance of nomograms

#### Internal Validation

The C-indexes for the nomogram of OS and CSS in the training cohort were 0.656[95% confidence interval (CI) 0.640-0.673)] and 0.666 (0.647-0.684), respectively, both of which were greater than the AJCC staging system [OS: 0.616(0.601-0.632); CSS: 0.616(0.598-0.645)].

In the validation cohort, the C-indexes for the new model of OS and CSS 0.648(0.623-0.673) and 0.655 (0.627-0.683), respectively, also presented superiority over the AJCC staging system [OS: 0.620 (0.597-0.643); CSS: 0.620 (0.593-0.647)].

In addition, we calculate the C-index of OS and CSS for the total data with the constructed nomograms and found that the C-index of OS is 0.653 [95% CI: 0.640-0.667], and the C-index of the corresponding stage is 0.617 (0.604-0.630); the C-index of CSS is 0.662 (0.647-0.678), and the value of the corresponding stage is 0.617 (0.602-0.632). All the results above indicate that our nomogram has obvious advantages over the traditional staging system. As far as we know, due to the rarity of OCS, the literatures on OCS are mainly based on sporadic case analysis, and there was no research on building nomograms based on large sample SEER database. However, to horizontal compare our model with the similar studies 10-14, we still compared and analyzed the independent prognostic risk factors included in other studies and included them in the SEER data of OS for C-index analysis **(Table 3).** We can note that, from the table, the C-index of our study is the highest in the current study on OCS.

**Figure 4:**
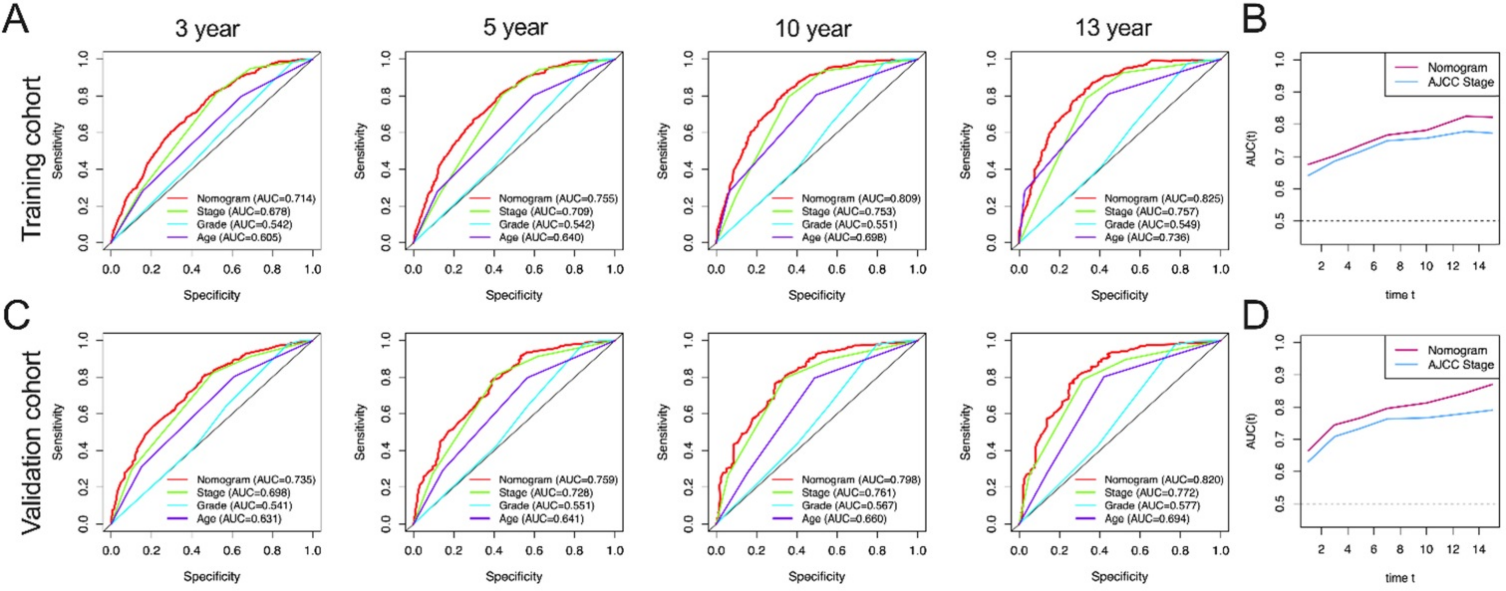
** AUC curves of the nomogram, AJCC stage, grade, and age for OS. (A)** AUC curves of the nomogram, AJCC stage, grade, and age in prediction of prognosis at 3-, 5-, 10- and13-year point in the training cohort. **(B)** Time dependent AUC curves of the nomogram and AJCC stage from 1 year to 16 year in the training cohort. **(C)** AUC curves of the nomogram, AJCC stage, grade, and age in prediction of prognosis at 3-, 5-, 10-and 13-year point in the validation cohort. **(D)** Time dependent AUC curves of the nomogram and AJCC stage from 1 year to 16 year in the validation cohort.

**Figure 5:**
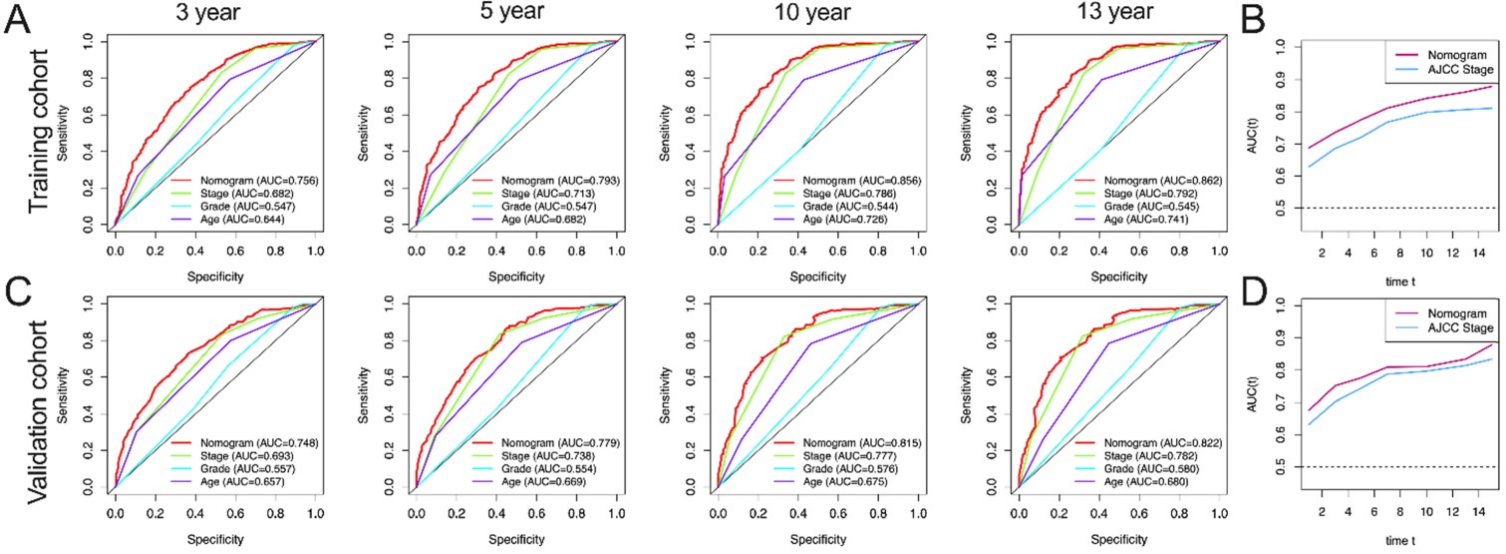
** AUC curves of the nomogram, AJCC stage, grade, and age for CSS. (A)** AUC curves of the nomogram, AJCC stage, grade, and age in prediction of prognosis at 3-, 5-, 10- and 13-year point in the training cohort. **(B)** Time dependent AUC curves of the nomogram and AJCC stage from 1 year to 16 year in the training cohort. **(C)** AUC curves of the nomogram, AJCC stage, grade, and age in prediction of prognosis at 3-, 5-, 10- and 13-year point in the validation cohort. **(D)** Time dependent AUC curves of the nomogram and AJCC stage from 1 year to 16 year in the validation cohort.

In this study, AUC in training group of OS nomograms, stage, grade and age of 3-, 5-, 10-, and 13-year were compared, respectively. We found that in OS, AUC of the 3-year, 5-year, 10 year and 13 year was 0.714, 0.755, 0.809 and 0.825 of nomogram in training group were significantly higher than that of stage (0.678, 0.709, 0.753 and 0.757), grade (0.542, 0.542, 0.551 and 0.549) and age (0.605, 0.640, 0.698 and 0.736) **(Figure 4A).** Similarly, AUC in training group of CSS nomograms, stage, grade and age of 3, 5, 10, and 13 year were also compared, respectively. We found that in CSS, AUC of the 3-year, 5-year, 10 year and 13 year was 0.756, 0.793, 0.856 and 0.862 of nomogram in training group were significantly higher than that of stage (0.682,0.713, 0.786, and 0.792), grade (0.547, 0.547, 0.544 and 0.545) and age ((0.644, 0.682, 0.726 and 0.741) **(Figure 5A).** Meantime, we also analyzed the AUC of 3, 5, 10, and 13 year of OS and CSS in validation cohorts, the results of validation group also showed that nomogram was better than stage, grade and age **(Figure 4C, and Figure 5C).** We also analyzed the time-dependent AUC of 3, 5, 10, and 13 year of OS and CSS in training and validation cohorts, the results of both groups showed that nomogram was better than stage **(Figure 4B, D and Figure 5B, D).**

**Figure 6:**
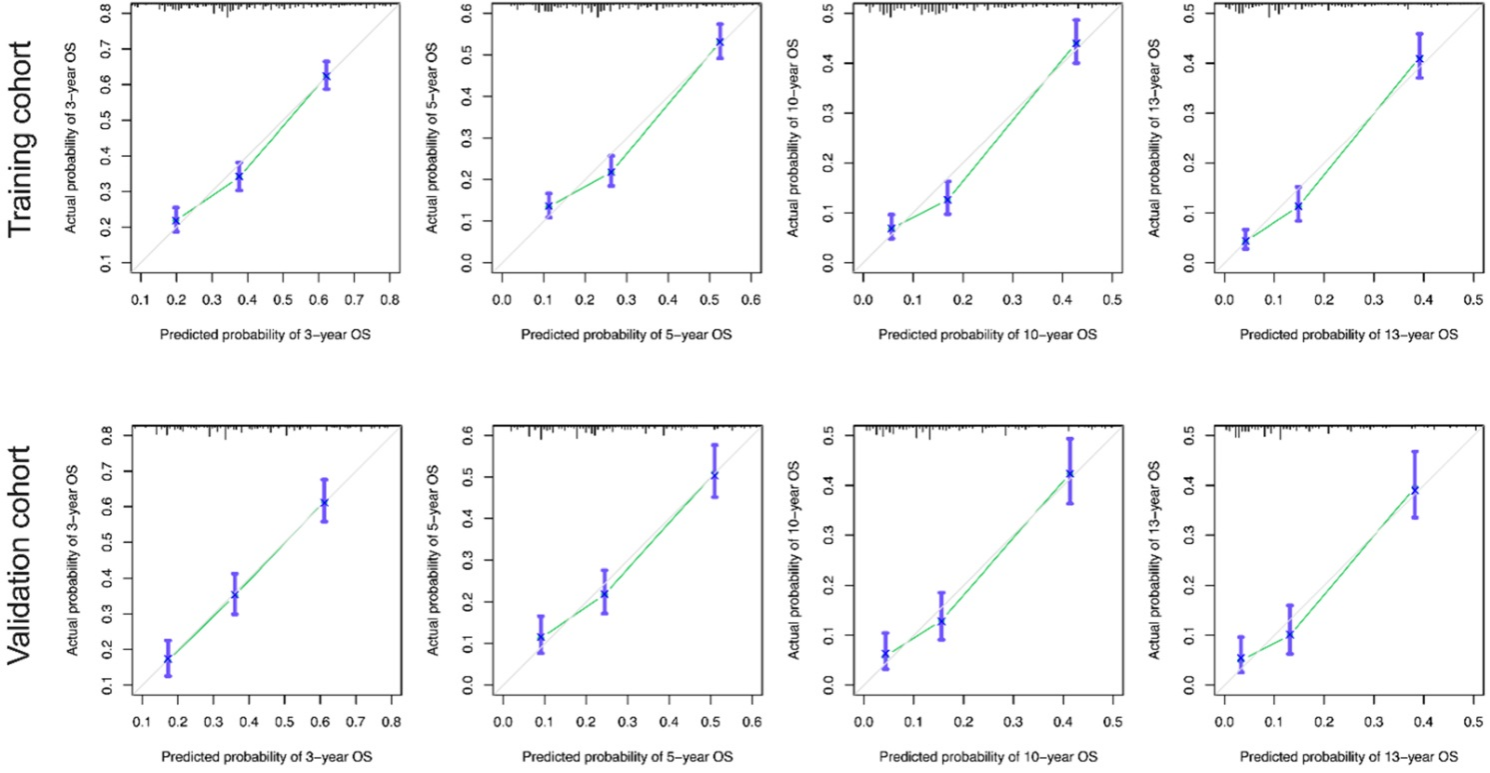
** Calibration curves for the OS nomogram. (A)** 3-, 5- , 10- and13-year calibration curves for the OS nomogram in the training cohort. **(B)** 3-, 5-, 10- and 13-year calibration curves for the OS nomogram in the validation cohort.

**Figure 7:**
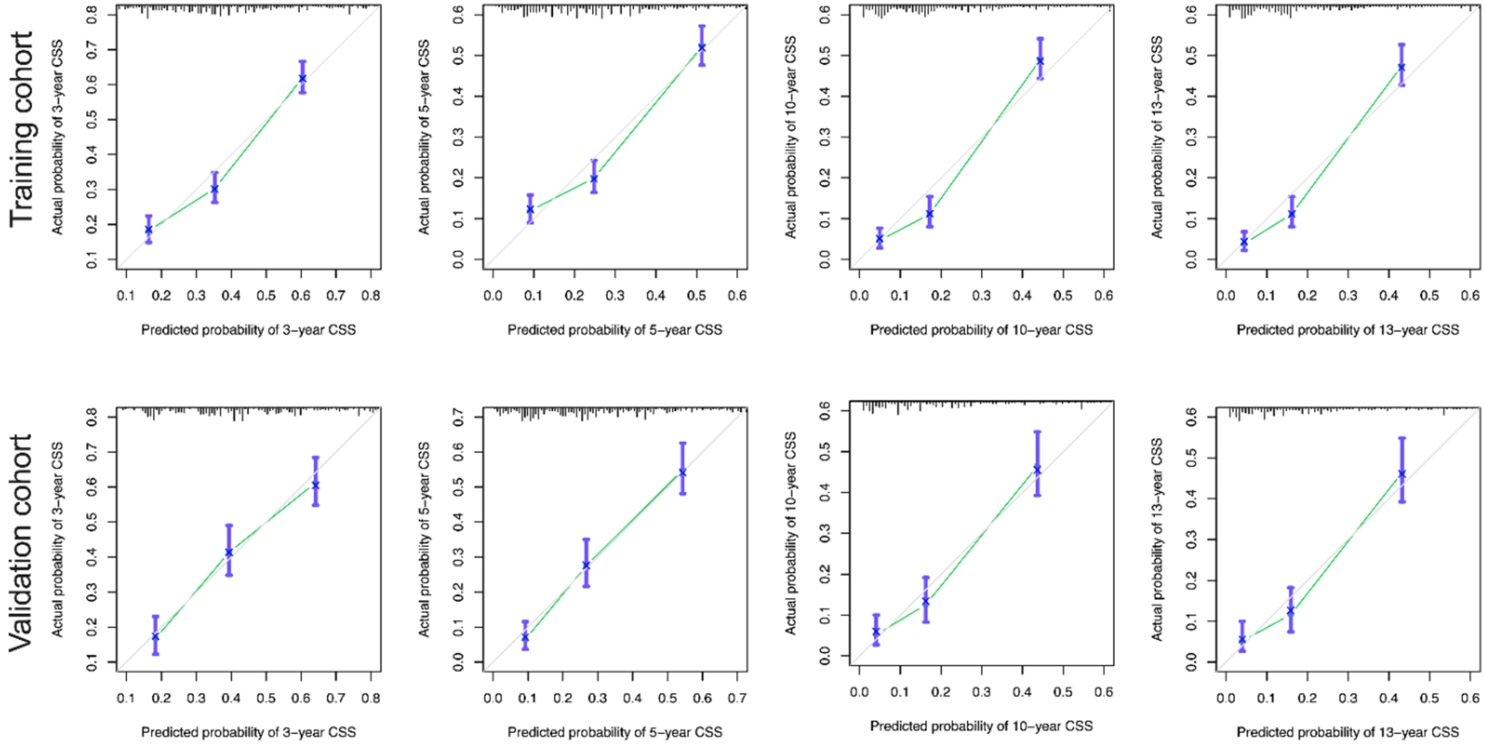
** Calibration curves for the CSS nomogram. (A)** 3-, 5-, 10- and 13-year calibration curves for the CSS nomogram in the training cohort. **(B)** 3-, 5-, 10- and 13-year calibration curves for the CSS nomogram in the validation cohort.

The calibration curves for the 3-, 5-, 10-, and 13-year OS were close to the gray line for the actual survival outcomes in the training cohort and validation cohort **(Figure 6).** Similarly, in CSS, in both training cohort and validation cohort, there is a good consistency between the predicted survival rate and the actual survival rate of nomogram **(Figure 7).**

**Figure 8:**
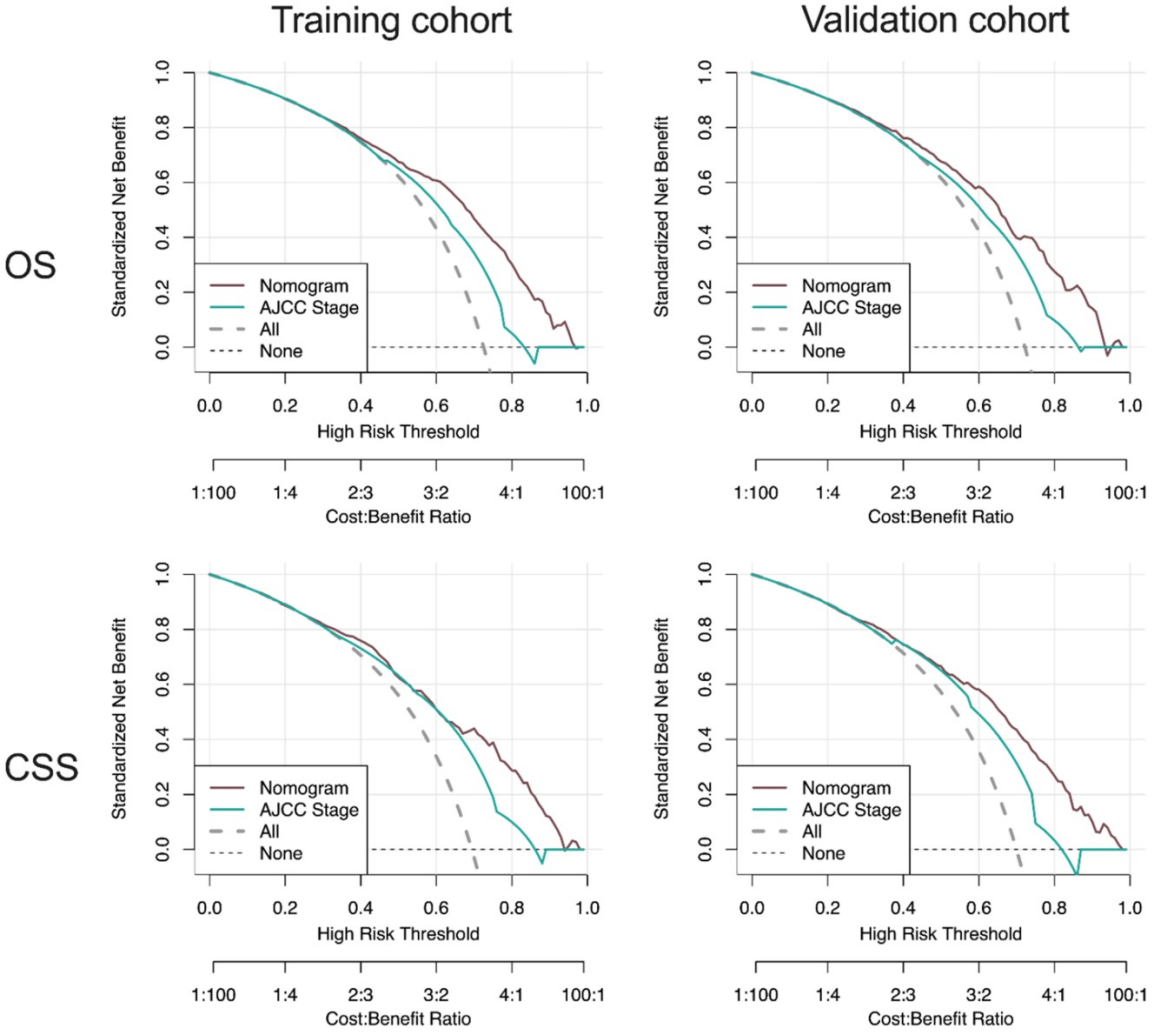
** DCA curve of the nomogram and AJCC stage for OS and CSS in the training and validation cohort.** DCA: decision curve analysis; AJCC: American Joint Commission on Cancer; OS: overall survival; CSS: cancer-specific survival.

Moreover, DCA curves indicated that the nomogram models, both the OS and CSS, made favorable predictions and outperformed than the stage system **(Figure 8).**

#### External Validation

The data of OCS (n=21) diagnosed in the Department of Gynecology, Shengjing Hospital of China Medical University from 2001 to 2021 were also analyzed. The last follow-up time was May 25, 2021. The statistical chart was shown in **Table 3**.

**Figure 9:**
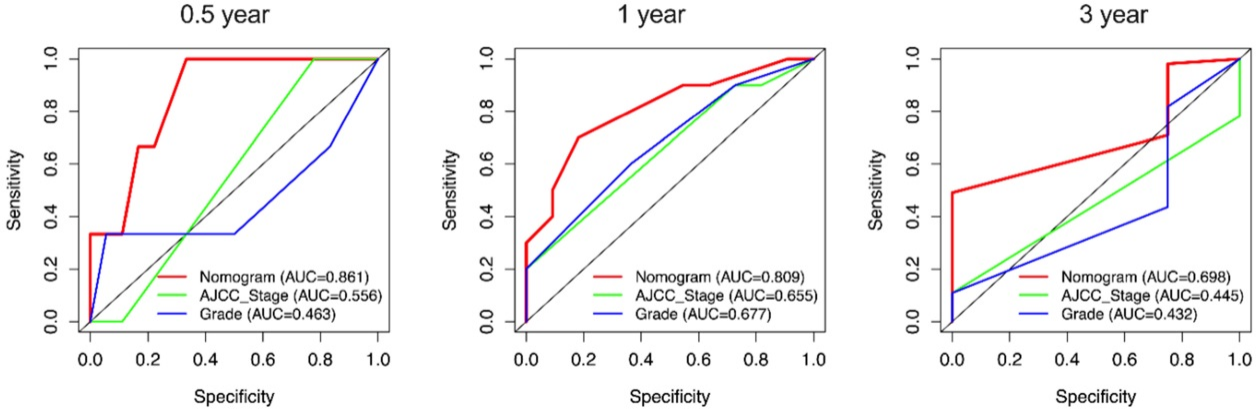
** External validation of the nomogram compared with AJCC stage in 21 cases of OCS from Shengjing Hospital.** AUC curves of the nomogram and AJCC stage in the prediction of prognosis at the 0.5-, 1-, and 3-year points.

Due to the rarity of carcinosarcoma, data of OCS from our hospital can only verify CSS. We found that in CSS, the C-indexes for the nomogram of CSS were 0.751 [95% confidence interval (CI): 0.652-0.851)] which was higher than the AJCC staging system 0.581(0.472-0.691). Furthermore, the AUCs of 0.5-, 1- and 3-year (0.861, 0.809 and 0.689) of nomogram were significantly higher than that of stage (0.556, 0.665 and 0.445) and grade (0.463, 0.677 and 0.432) **(Figure 9)**.

The above internal and external validation results indicate that our nomogram has better performance.

## Discussion

Due to the rarity of OCS, a large sample of cases for clinical research is lacking, but the SEER database provides effective clinical data for research on rare kinds of tumors. Based on SEER data, we identified five independent prognostic risk factors for OS and seven independent prognostic risk factors for CSS in patients with OCS using univariable and multivariable analyses. Based on these results, nomograms of OS and CSS for OCS were constructed. After verification, we concluded that the models had good discriminatory and calibration capabilities and thus, could be used as practical tools for the clinical evaluation of prognosis in patients with OCS.

The staging system is a traditional tool for evaluating tumor prognosis. Most studies have shown that stage is an independent factor for the OS and CSS of patients with OCS 12, 15, while some studies have shown that stage was not associated with OCS prognosis 16. The reason for this discrepancy may be the small number of cases, most of which involved advanced stage disease; the rarity of early-stage cases could have reduced the statistical power of the reported case series 17. In the present study, among the 2,753 patients included, stage I-II (early stage) disease accounted for 30.2% of all cases, and stage III-IV (late stage) disease accounted for 69.8% of all cases, consistent with the actual distribution of clinical patients in real-world settings; this enhances the credibility of the models presented here. This study indicated that stage was the most significant prognostic factor for the OS and CSS of patients with OCS, and an advanced stage tended to be associated with a worse prognosis.

OCS often occurs in older women with an average age of 60-70 years. Although published prognostic analyses of other pathological types of ovarian epithelial carcinoma have indicated that age is an important factor related to OS, the significance of age in evaluating the prognosis of patients with OCS remains controversial 13, 18. In the present study, we used X-tile analysis to derive the cutoff points for age: 57 and 74 years for OS and CSS which made the assessment of the correlation between age and OCS prognosis more accurate. Through univariable and multivariable analyses, we concluded that age is an independent prognostic factor for both OS and CSS in patients with OCS. Older patients are more likely to show worse survival outcomes, which may be due to a lower immune response 19.

We found that grade was an important index for evaluating not only the degree of malignancy but also the prognosis of ovarian cancer. Grade has been found to be associated with the prognosis of OCS in many studies 20. Paulsson et al. found that the grade (i.e., grade 3 vs. 1-2) of the epithelial component of the tumor was a significant prognostic factor using multivariable Cox analysis 17. The present study confirmed that grade is an important independent factor for OS and CSS in patients with OCS. A higher grade tended to be associated with a worse prognosis. We also found that tumor size was an independent factor for CSS in patients with OCS. Tumor size might reflect the degree of malignancy of the tumor, and a higher degree of malignancy leads to a faster growth rate and a worse prognosis.

Previous studies have reported that compared to serous ovarian cancer, OCS is likely to occur in unmarried women 21. The cited study further found that among OCS patients, marital status was correlated with better CSS. The reason for this might be that unmarried or single patients tend to opt for fertility-preserving surgery that fails to achieve optimal cytoreduction, leading to relapse.

Because of its rarity, there is no standardized treatment plan for OCS. In clinical practice, OCS is generally treated using the same approach as that for other epithelial ovarian cancers, i.e., with maximal cytoreductive surgery followed by chemotherapy 22, 23. Studies have found that OCS has the pathological characteristics of both carcinoma and sarcoma and a biological behavior closer to that of epithelial ovarian cancer, which is characterized by lymphatic rather than hematogenous metastasis. Therefore, lymph node dissection is often performed for treating OCS. A study by Garg et al. 14 investigated the association of lymphadenectomy with survival, and the results indicated that lymph node dissection was an independent prognostic factor for CSS in patients with OCS. In a series of more than 900 patients, slightly more than 40% of patients underwent extended surgery with lymphadenectomy. The risk of death was reduced by 34% after lymphadenectomy, compared to that with no lymphadenectomy 24. We also analyzed the correlation between regional lymph node dissection and the prognosis of OCS in the present study. We found that the smaller the region of lymph node dissection, the worse the CSS. These results suggest that lymph node dissection is necessary for the treatment of OCS. Wang et al. found that worse survival was observed in the lymph node (LN) (+) group than in the LN (-) group among patients with advanced OCS 20. In the present study, using X-tile analysis, we adopted 9 and 10 as thresholds for the number of lymph node metastases for OCS prognosis analysis; we found that the number of lymph node metastases was an independent factor for OS. The greater the number of lymph node metastases, the worse the prognosis.

In the treatment of ovarian cancer, the status of residual pelvic lesions is an important indicator of prognosis and guides follow-up treatment 25, 26. Rauh-Hain et al. 27 found that patients with OCS with only microscopic disease after cytoreduction had a median OS of 47 months, compared to a median OS of 18 months in those with optimal but macroscopic disease and of 8 months in those with suboptimal disease after surgery. Our study reached a similar conclusion: residual lesions larger than 1 cm after surgery were independent prognostic factors for both OS and CSS, which indicates that minimizing residual lesions during surgery plays an important role in improving the prognosis of OCS.

Most studies indicate that both the CSS and OS of patients with OCS are shorter than those of patients with serous ovarian carcinoma across all stages 6. Another study showed that the OS rates of patients with OCS and high-grade serous ovarian carcinoma seemed to be similar when optimal cytoreduction was achieved and followed by platinum-taxane combination chemotherapy 28. Paulsson et al. 14 concluded that adjuvant therapy and six completed cycles of chemotherapy were the most important prognostic factors for OCS. In the present study, we analyzed the relationship between chemotherapy and OCS prognosis and found no correlation. This might be due to the fact that the SEER database lacks data on chemotherapy treatments and patterns, which made it impossible to further quantify the effect of the chemotherapy regimen and treatment course on the prognosis of OCS.

To our knowledge, this is the first study to use the nomogram for predicting the prognosis of OCS, though some literatures have studied the correlation between different factors and the prognosis of OCS. The C-index of our nomogram was higher than those reported by other studies 10-14. Importantly, external validation was performed using data from 21 eligible patients with OCS. The C-index and AUC results also indicated that the nomogram exhibited excellent performance, though because of the rarity of OCS, data of OCS from our hospital could only verify CSS.

This study has some limitations. First, similar to that in all research based on the SEER database, selection bias was inevitable due to the study's retrospective nature. Second, it was not possible to analyze the OCS tumors for homozygosity or heterozygosity; this information would have been valuable because the heterologous subtype is known to be associated with poor disease-free survival and OS 16.

## Conclusions

In this study, we constructed nomograms using the SEER database to predict the 3-, 5-, 10-, and 13-year OS and CSS rates in patients with OCS. The nomograms were internally and external verified using the SEER database and externally validated in patients with OCS treated at Shengjing Hospital of China Medical University and were found to have good predictive ability and clinical application value. In addition, these nomograms can serve to stratify patients by risk to support more personalized treatment and follow-up, ultimately improving the survival rate. To our knowledge, our nomograms are currently the best and most directly applicable models for predicting OS and CSS in patients with OCS, and these nomograms may also be useful for providing prognostic information in clinical settings.

## Figures and Tables

**Table 1:** Patients' demographics and clinicopathological characteristics

Variables	N (%)	Mean survival time mean (95%CI) months	Median survival time median (95%CI) months	Training set [n(%)]	Validation set [n(%)]	P
Total	2753			1929 (70.0)	824 (30.0)	
**Year of diagnosis**						0.667
1988-1990	130 (4.7)	23 (19-27)	17 (12-25)	87 ( 4.5)	43 ( 5.2)	
1991-2000	686 (24.9)	26 (24 -28)	21 (19-26)	473 (24.5)	213 (25.8)	
2001-2010	1028 (37.3)	29 (27-31)	23 (20-26)	722 (37.5)	306 (37.2)	
2011-2016	909 (33.1)	34 (32-36)	23(21-27)	647 (33.5)	262 (31.8)	
**Age, years**						0.367
≤56	725 (26.4)	40 (37-43)	50 (41-66)	503 (26.0)	222 (26.9)	
57-74	1374 (49.9)	30 (29-32)	22 (20-25)	979 (50.8)	395 (47.9)	
75-101	654 (23.7)	22 (20-23)	13 (12-14)	447 (23.2)	207 (25.2)	
**Race**						0.535
White	2344(85.1)	30 (29-31)	22 (21-25)	1642 (85.1)	702 (85.2)	
Black	214 (7.8)	27 (23-32)	16 (14-21)	145 (7.5)	69 (8.4)	
Others	195 (7.1)	39 (33-44)	47 (23-67)	142 (7.4)	53 (6.4)	
**Insure**						0.827
Unknown	1497 (54.4)	25 (24 -27)	21 (19-24)	1044 (54.1)	453 (55.0)	
Insured	1226 (44.5)	38 (36-41)	23 (21-27)	865 (44.8)	361 (43.8)	
Uninsured	30 (1.1)	28 (19-38)	230 (15-48)	20 (1.0)	10 (1.2)	
**Marriage**						0.834
Single	391 (14.2)	34 (30-38)	24 (20-31)	270 (14.0)	121 (14.7)	
Married	1448 (52.6)	31 (30-33)	26 (23-28)	1013 (52.5)	435 (52.8)	
Widowed/Separated/ Divorced/ Unknown	559 (20.3)	27 (25 -29)	17 (16-21)	646 (33.5)	268 (32.5)	
**Lateral**						0.675
Unilateral	1760 (63.9)	33 (31 -35)	26 (23-30)	1233 (63.9)	527 (64.0)	
Paired	107 (3.9)	19 (15-23)	9 (7-18)	79 (4.1)	28 (3.4)	
Bilateral	886 (32.2)	27 (25-28)	20 (17-22)	617 (32.0)	269 (32.6)	
**Tumor size(mm)**						0.204
≤210	1003 (36.4)	38 (36-41)	25 (22-28)	723 (37.5)	280 (34.0)	
>210	107 (3.9)	27 (20-34)	13 (10-16)	73 (3.8)	34 (4.1)	
Unknown	1643 (59.7)	26 (25-28)	17 (16-18)	1133 (58.7)	510 (61.9)	
**Preoperative serum CA125 level**						0.294
Negative/Borderline	112 (4.1)	51 (43-60)	53 (32-NA)	74 (3.8)	38 (4.6)	
Positive	1097 (39.8)	34 (32-37)	23 (21-26)	785 (40.7)	312 (37.9)	
Unknown	1544 (56.1)	27 (26-28)	21 (19-23)	1070 (55.5)	474 (57.5)	
**Surgery for primary lesions**						0.544
Unknown	576 (20.0)	25 (23-27)	20 (17-24)	394 (20.5)	182 (22.1)	
Furtile	170 (6.2)	36 (31-42)	29 (21-51)	112 (5.8)	58 (7.0)	
Not furtile	753 (27.3)	39 (36-41)	39 (32-47)	535 (27.7)	218 (26.5)	
Debulking	1190 (43.2)	27 (26-29)	18 (16-20)	841 (43.6)	349 (42.4)	
Pelvic exenteration	64 (02.3)	28 (22-35)	26 (14-45)	47 (2.4)	17 (2.1)	
**Regional LN dissected**						0.718
Undo	803 (29.1)	28 (26-30)	17 (15-19)	574 (29.8)	229 (27.8)	
1-3	234 (08.5)	33 (29-38)	23 (18-30)	164 ( 8.5)	70 ( 8.5)	
4 or more reg	599 (21.8)	44 (41-48)	39 (32-46)	420 (21.7)	179 (21.7)	
Unknown	1117 (40.6)	26 (24 -27)	21 (18-23)	771 (40.0)	346 (42.0)	
**LN examined**						0.834
Undo	1494 (54.2)	25 (24-27)	16 (15-19)	1055 (54.7)	439 (53.3)	
≤3	148 (05.4)	29 (24-34)	17 (15-24)	106 (5.5)	42 (5.1)	
≥4	996 (36.2)	39 (36-41)	37 (32-46)	688 (35.7)	308 (37.4)	
Unknown	115 (04.2)	28 (23-33)	21 (14-31)	80 (4.1)	35 (4.2)	
**LN positive**						0.540
Undone	1494 (54.2)	25 (24-27)	16 (15-19)	1055 (54.7)	439 (53.3)	
Negative	832 (30.2)	40 (38-43)	46 (37-56)	577 (29.9)	255 (30.9)	
≤9	342 (12.4)	32 (28-36)	25 (20-30)	244 (12.6)	98 (11.9)	
≥10	34 (1.2)	20 (12-29)	9 (6-28)	21 (1.1)	13 (1.6)	
Unknown	51 (2.0)	20 (14-27)	11 (8-21)	32 (1.7)	19 (2.2)	
**Grade**						0.849
I	61 (2.2)	60 (49-71)	NA (172-NA)	41 (2.1)	20 (2.4)	
II	90 (3.3)	36 (29-43)	105 (44-NA)	60 (3.1)	30 (3.6)	
III	888 (32.2)	31 (29-33)	23 (21-26)	624 (32.3)	264 (32.0)	
IV	571 (20.6)	30 (27-32)	18 (16-22)	408 (21.2)	163 (19.8)	
Unknown	1143 (41.4)	28 (26-30)	20 (19-24)	796 (41.3)	347 (42.1)	
**AJCC Stage**						0.408
I	437 (15.9)	51 (47-55)	199 (149-289)	295 (15.3)	142 (17.2)	
II	395 (14.3)	36 (32-40)	34 (29-45)	288 (14.9)	107 (13.0)	
III	1308 (47.5)	27 (25-28)	18 (16-20)	916 (47.5)	392 (47.6)	
IV	613 (22.3)	22 (20-24)	14 (12-15)	430 (22.3)	183 (22.2)	
**Residual lesion size**						0.643
No residual lesion	551 (20.0)	42 (38-44)	31 (25-41)	393 (20.4)	158 (19.2)	
≤1 cm	104 (3.8)	22 (19-29)	15 (12-22)	68 (3.5)	36 (4.4)	
>1 cm	56 (2.0)	28 (20 -36)	18 (12-40)	101 (5.2)	46 (5.6)	
Residual unknown	2042 (74.2)	27 (20 -34)	18 (14-24)	1367 (70.9)	584 (70.9)	
**Radiotherapy**						0.427
None/Unknown	2634 (95.7)	30 (29-32)	23 (21-25)	1850 (95.9)	784 (95.1)	
Yes	119 (04.3)	26 (21-31)	16 (14-23)	79 (4.1)	40 (4.9)	
**Chemotherapy**						0.663
None/Unknown	796(28.9)	27 (24-29)	15 (12-23)	563 (29.2)	233 (28.3)	
Yes	1957(71.1)	32 (30-33)	23 (22-25)	1366 (70.8)	591 (71.7)	
**Organ metastasis**						0.128
Bone						
None/Unknown	2747 (99.8)	30 (29-31)	22 (21-24)	1927(99.9)	820 (99.5)	
Yes	6 (0.2)	25 (1-49)	9 (7-NA)	2 (0.1)	4 (0.5)	
**Brain**						0.879
None/Unknown	2751 (99.9)	30 (29 -31)	22 (21-24)	1927 (99.9)	824 (100.0)	
Yes	2 (00.1)	12 (12-12)	12(12-NA)	2 (0.1)	0 (0.0)	
**Liver**						1.000
None/Unknown	2697 (98.0)	30 (29-32)	22 (21-25)	1890 (98.0)	807 (97.9)	
Yes	56 (2.0)	25 (18-31)	14 (12-24)	39 (2.0)	17 (2.1)	
**Lung**						0.498
None/Unknown	2705 (98.3)	30 (29-31)	22 (21-24)	1898 (98.4)	807 (97.9)	
Yes	48 (1.7)	29 (21-37)	21 (15-33)	31 (1.6)	17 (2.1)	

**Table 2:** Univariate and Multivariate Cox analysis

Variables	OS				CSS			
	Univariate analysis		Multivariate analysis		Univariate analysis		Multivariate analysis	
	HR (95%CI)	*P*	HR (95%CI)	*P*	HR (95%CI)	*P*	HR (95%CI)	*P*
**Year of diagnosis**	0.985 (0.924-1.049)	0.631	-		0.911 (0.846-0.980)	0.01		
1988-1990							Reference	
1991-2000							0.798 (0.584-1.091)	0.157
2001-2010							0.764 (0.506-1.153)	0.200
2011-2016							0.605 (0.382-0.960)	0.053
**Age, years**	1.675 (1.550-1.809)	<0.001			1.784 (1.631-1.951)	<0.001		
≤56			Reference				Reference	
57-74			1.422 (1.229-1.646)	<0.001			1.456 (1.234-1.719)	<0.001
74-101			2.210 (1.869-2.613)	<0.001			2.288 (1.888-2.773)	<0.001
**Race**	0.931 (0.845-1.025)	0.146	-		0.892 (0.797-0.998)	0.46	-	
White								
Black								
Others								
**Insure**	0.962 (0.864-1.071)	0.479	-		0.909 (0.804-1.028)	0.128	-	
Unknown								
Insured								
Uninsured								
**Marriage**	1.263 (1.162-1.374)	<0.001			1.307 (1.184-1.443)	<0.001		
Single			Reference				Reference	
Married			0.811 (0.682-0.964)	0.018			0.787 (0.645-0.961)	0.019
Widowed/Separated/Divorced/ Unknown			0.958 (0.797-1.152)	0.648			0.979 (0.792-1.210)	0.843
**Lateral**	1.177 (1.113-1.244)	<0.001			1.162 (1.090-1.239)	<0.001	-	
Unilateral			Reference					
Paired			1.172 (0.892-1.539)	0.254				
Bilateral			1.015 (0.985-0.903)	0.799				
**Tumor size (mm)**	1.041 (0.983-1.102)	0.168			1.079 (1.011-1.152)	0.023		
≤210							Reference	
>210							1.644 (1.176-2.299)	0.004
Unknown							1.069 (0.878-1.301)	0.506
**Preoperative serum CA125 level**	1.1.063 (0.967-1.169)	0.208	-		1.127 (1.010-1.257)	0.033	-	
Negative/Borderline							1.085 (0.718-1.639)	0.699
Positive							1.080 (0.702-1.661)	0.727
Unknown					NA	NA	NA	NA
**Surgery for primary lesions**	1.103 (1.053-1.155)	<0.001			1.066 (1.010-1.125)	0.020		
Unknown			Reference				Reference	
Furtile			0.983 (0.745-1.295)	0.901			0.970 (0.665-1.417)	0.876
Not furtile			0.945 (0.788-1.134)	0.544			0.924 (0.701-1.217)	0.571
Debulking			1.089 (0.917-1.293)	0.332			1.056 (0.815-1.369)	0.679
Pelvic exenteration			0.975 (0.685-1.389)	0.890			0.855 (0.547-1.336)	0.490
**Regional LN dissected**	0.906 (0.868-0.947)	<0.001			0.934 (0.888-0.982)	0.008	-	
Undo			Reference				Reference	
1-3			1.052 (0.795-1.391)	0.724			1.003 (0.718-1.399)	0.988
4 or more reg			0.789 (0.627-0.994)	0.044			0.762 (0.583-0.995)	0.046
Unknown			0.910 (0.771-1.075)	0.268			0.872 (0.666-1.143)	0.321
**LN examined**	0.812 (0.770-0.856)	<0.001			0.799 (0.751-0.851)	<0.001		
Undo			Reference				Reference	
≤3			1.675 (0.937-2.994)	0.082			0.676 (0.394-1.160)	0.326
≥4			1.435 (0.845-2.437)	0.181			0.525 (0.312-0.884)	0.430
Unknown			1.306 (0.886-1.926)	0.178			0.763 (0.519-1.122)	0.217
**LN positive**	0.884 (0.825-0.948)	<0.001			0.882 (0.815-0.954)	0.002	-	
Undone			Reference				Reference	
Negative			0.511 (0.309-1.845)	0.009			0.591 (0.318-1.100)	0.097
≤9			0.501 (0.296-0.845)	0.010			0.574 (0.302-1.090)	0.090
≥10			0.993 (0.491-2.001)	0.983			1.146 (0.516-2.543)	0.739
Unknown			NA	NA			NA	NA
**Grade**	1.120 (1.067-1.176)	<0.001			1.119 (0.057-1.186)	<0.001		
I			Reference				Reference	
II			1.714 (0.894-3.287)	1.105			1.809 (0.726-4.504)	0.203
III			2.185 (1.243-3.840)	0.007			2.454 (1.076-5.602)	0.033
IV			2.308 (1.305-4.082)	0.004			2.654 (1.156-6.092)	0.021
Unknown			2.224 (1.270-3.896)	0.005			2.435 (1.070-5.543)	0.034
**AJCC Stage**	1.601 (1.513-1.694)	<0.001			1.680 (1.569-1.799)	<0.001		
I			Reference				Reference	
II			2.317 (1.825-2.942)	<0.001			3.204 (2.291-4.483)	<0.001
III			3.493 (2.801-4.355)	<0.001			5.100 (3.730-6.973)	<0.001
IV			3.765 (2.988-4.745)	<0.001			5.362 (3.882-7.406)	<0.001
**Residual lesion size**	1.049 (1.012-1.088)	0.009			1.052 (1.011-1.096)	0.014	-	
No residual lesion			Reference				Reference	
≤1 cm			1.382 (0.997-1.916)	0.052			1.074 (0.668-1.727)	0.769
>1 cm			1.016 (0.675-1.527)	0.941			1.502 (1.054-2.142)	0.024
Residual unknown			1.328 (1.119-1.576)	0.001			1.170 (0.930-1.472)	0.179
**Radiotherapy**	1.049 (0.808-1.362)	0.718	-		1.029 (0.761-1.392)	0.852	-	
None/Unknown								
Yes								
**Chemotherapy**	0.973 (0.865-1.094)	0.644	-		0.0991 (0.861-1.140)	0.896	-	
None/Unknown								
Yes								
**Organ metastasis**	0.482 (0.068-3.424)	0.466	-		1.248 (0.176-8.870)	0.824	-	
Bone								
None/Unknown								
Yes								
**Brain**	1.217 (0.171-8.655)	0.844	-		1.176 (0.165-8.362)	0.872	-	
None/Unknown								
Yes								
**Liver**	1.177 (0.772-1.794)	0.450	-		1.143 (0.717-1.824)	0.574	-	
None/Unknown								
Yes								
**Lung**	1.009 (0.641-1.587)	0.970	-		0.909 (0.546-1.514)	0.713	-	
None/Unknown								
Yes								

**Table 3:** Comparison of C-indexes

	Cases		C index (95%CI)
Seer-based nomograms	2753	OS Nomogram	0.653 (0.640-0.667)
		OS Stage	0.617 (0.604-0.630)
	2112	CSS Nomogram	0.662 (0.647-0.678)
		CSS Stage	0.617 (0.602-0.632)
Vickers AJ et al. 10	40	Stage+Chemo+Surgery	0.644 (0.631-0.658)
Cicin I et al. 11	26		
Chun KC et al. 12	81	Stage+Chemo	0.643 (0.630-0.657)
Nizam A et al. 13	363	Stage+Regional_LN_scope	0.622 (0.613-0.640)
Garg G et al. 14	51	Residual lesion size	0.520 (0.510-0.531)

## Abbreviations

CSS: Cancer-specific survival
OCS: Ovarian carcinosarcoma
OS: Overall survival
SEER: Surveillance, Epidemiology, and End Results
LN: Lymph node
LASSO: Least absolute shrinkage and selection operator
